# Powered Haulage Fatalities in Appalachian Coal Mines

**DOI:** 10.13023/jah.0604.02

**Published:** 2025-01-29

**Authors:** Nirmala T. Myers, Noemi B. Hall, Nadia T. Saif, A. Scott Laney

**Affiliations:** CDC NIOSH; CDC NIOSH; CDC NIOSH; CDC NIOSH

**Keywords:** Appalachia, Appalachian coal mines, fatalities, powered haulage

## Abstract

The recent death of a 33-year-old mother of three at a Central Appalachian surface coal mine highlights the persistent dangers faced by miners, particularly from powered haulage incidents. As of December 2, 28 mining fatalities have occurred in the U.S. in 2024, with four in Appalachian coal mines attributed to powered haulage. These deaths underscore the urgent need to address this cause of fatalities in the mining industry and to provide a safe workplace to protect all miners.

Aminer from Erbacon, West Virginia passed away on Tuesday, July 23, 2024, after injuries sustained at a Central Appalachian surface coal mine eleven days prior.^1^,^2^ The 33-year-old mother of three was a rock truck operator at the Pocahontas Wyco surface mine in Helen, West Virginia. The Mine Safety and Health Administration (MSHA) published a preliminary report^3^, a fatality alert^4^, and recently a final report^5^ describing the incident: “On July 12, 2024, a rock truck operator was seriously injured after being struck by the bucket of a front-end loader. While walking to her parked truck, the rock truck operator passed under the raised front-end loader bucket as it was being lowered to the ground during maintenance.”

In the subsequent months, there were seven more miners fatally injured in incidents related to powered haulage – the use of equipment, including belt conveyers, bulldozers, service trucks, front-end loaders, and other large vehicles to transport materials and people in surface and underground mines. Two of them occurred in coal miners in Appalachia, a region encompassing states as designated by the Appalachian Regional Commission.^6^

Since 2001, 417 of the 508 fatal injuries sustained by US coal miners (82.1%) have occurred in Appalachian coal mines (274 underground and 143 surface). Though mine disasters (mass casualty explosions, collapses) garner public attention, incidents involving powered haulage accounts for the majority of deaths in Appalachian coal mines. For women coal miners since 1979, there have been 11 fatalities, six by powered haulage and of those, five in Appalachian coal mines.

Solely in 2024, (as of December 2) there have been 28 mining fatalities in the U.S. – seven out of 18 fatalities in metal/nonmetal mines (38.9%) and five out of 10 fatalities in coal mines (50.0%) were classified as “powered haulage.” Four out of the five coal fatalities were in the Appalachian Region. Though the coal mining workforce has decreased by half in recent years (from 143,437 coal miners in 2011 to 68,631 coal miners in 2023), the rate of mining fatalities per 100,000 coal miners has not declined significantly (*Joinpoint Regression Program, Version 5.2.0.0. April, 2024)*. The rate of coal fatalities in 2011 was 13.9 per 100,000 coal miners, whereas in 2023, it was 13.1 per 100,000 coal miners.^7^ In 2018, MSHA posted a Request for Information (RFI)^8^ and held a series of public meetings along with other initiatives to reduce accidents caused by powered haulage. Remaining a targeted safety and health initiative,^9^–^11^ MSHA recently published a final rule, Safety Program for Surface Mobile equipment, to fortify existing safety programs for powered haulage and to improve safety around the use of all surface mobile equipment at mine sites. The July 12^th^ incident that ultimately led to the aforementioned miner’s death occurred just five days before MSHA began enforcement of this standard on July 17, 2024.^12^ Unfortunately, each fatality attributable to powered haulage underscores the need for this new regulation and for ongoing vigilance with regards to powered haulage safety.

In our work at the National Institute for Occupational Safety and Health (NIOSH), an institute of the Centers for Disease Control and Prevention (CDC), we work towards eliminating mining fatalities, injuries, and illnesses through relevant research and impactful solutions. The miner who died following the incident on July 12 was an Appalachian woman with a young family. In addition to our research focusing on women in the mining workforce,^13^,^14^ some of our current efforts in improving powered haulage safety are collision avoidance technologies, simulator-based trainings, and emergency response planning.^15^ As the average American spends half of their waking hours at work, work is an important component of helping young families thrive and constitutes an important contribution to not only household economics but also to overall health.^16^

It is crucial to prioritize miners’ safety and health by providing appropriate training and ensuring safe working conditions. Some key actions to enhance safety measures and to reduce powered haulage-related fatalities are ensuring adequate lighting, reflective wear, and safe working space during machinery or equipment maintenance and good communication between operators.^4^,^5^ Emergency response procedures and conducting regular drills can ensure miners are familiar with their equipment and are prepared to handle unexpected events promptly and efficiently. While there have been significant strides in reducing mining fatalities over the years, more efforts are necessary to further lower the number of occupational deaths. MSHA’s rule is a first step in changing the safety culture surrounding these types of incidents, which will then empower miners to protect themselves and others on the job.
